# Supplementary material for: Photoinitiated Cationic Ring-Opening Polymerization of Octamethylcyclotetrasiloxane
Source: Molecules. 2023 Jan 29;28(3):1299. doi: 10.3390/molecules28031299 (PMC9919424; doi:10.3390/molecules28031299)
Supplement: Supplementary file 1 [file molecules-28-01299-s001.zip › molecules-2131362-supplementary.pdf]

Communication

Photoinitiated Cationic Ring-Opening Polymerization of Octamethylcyclotetrasiloxane

Zehra Gul Coban, Huseyin Cem Kiliclar and Yusuf Yagci *

Department of Chemistry, Istanbul Technical University, 34469 Maslak, Turkey; cobanz19@itu.edu.tr (Z.G.C.); kiliclar@itu.edu.tr (H.C.K.)
* Correspondence: yusuf@itu.edu.tr; Tel.: +90-212-285-32-41

Details of the matematical modelling in Figure 2

a

Model	Asymptotic1
Equation	y = a-b*c^x
Plot	reduced visc.
a	9,33997 ± 1,1793
b	9,94589 ± 1,86916
c	0,81979 ± 0,06821
Reduced Chi-Sqr	2,56405
R-Square(COD)	0,90433
Adj. R-Square	0,84055

b

Model	Asymptotic1
Equation	y = a-b*c^x
Plot	reduced visc.
a	7,82838 ± 1,24588
b	8,13362 ± 1,52971
c	0,88566 ± 0,05236
Reduced Chi-Sqr	1,60887
R-Square(COD)	0,90693
Adj. R-Square	0,84488

c

Model	Asymptotic1
Equation	y = a-b*c^x
Plot	reduced visc.
a	8,34118 ± 0,29399
b	8,3699 ± 0,48798
c	0,79125 ± 0,02419
Reduced Chi-Sqr	0,17573
R-Square(COD)	0,98993
Adj. R-Square	0,98321

d

Model	Asymptotic1
Equation	y = a-b*c^x
Plot	reduced viscosity
a	8,8086 ± 1,10594
b	9,07899 ± 1,21662
c	0,90038 ± 0,03501
Reduced Chi-Sqr	0,93036
R-Square(COD)	0,95339
Adj. R-Square	0,92232

Figure S1. Details of exponential curve fitting for (a) PDMS-1, (b) PDMS-2, (c) PDMS-3 and (d) PDMS-4.
